# Immune activation and MRgFUS

**DOI:** 10.1186/2050-5736-3-S1-O41

**Published:** 2015-06-30

**Authors:** Katherine Ferrara, Elizabeth Ingham, Andrew Wong, Azadeh Kheirolomoom, Brett Fite, Yu Liu, Lisa Mahakian, Sarah Tam

**Affiliations:** 1 University of California Davis, Davis, California, United States

## Background/introduction

For many years, immune activation following tumor ablation has been evaluated in the treatment of systemic cancer. Ultrasound ablation is thought to promote dendritic cell maturation and T-cell immunity, and is particularly advantageous because it is non-invasive, can be controlled with high spatial precision and uses no harmful ionizing radiation.

## Methods

Recently, immune adjuvants have been shown to be effective in treating metastatic cancer, with cancer immunotherapy named as the “breakthrough of the year” in 2013. At this time, combining ablation with immune adjuvants is a promising technique for expanding the utility of ultrasound for the treatment of systemic disease. We will briefly review the status of this combined therapy and opportunities for future studies.

